# Detection of Paratuberculosis in Breeding Bulls at Pakistani Semen Production Units: A Continuous Source of Threat

**DOI:** 10.5402/2011/501235

**Published:** 2011-12-22

**Authors:** Muhammad Abbas, Muhammad Munir, Syed Abdul Khaliq, Muhammad Ikram Ul Haq, Muhammad Tanveer Khan, Zafar ul Ahsan Qureshi

**Affiliations:** ^1^Quality Control Laboratory, Veterinary Research Institute, Lahore, Pakistan; ^2^Department of Biomedical Sciences and Veterinary Public Health, Swedish University of Agricultural Sciences, 750 07 Uppsala, Sweden; ^3^Department of Natural Sciences, University of Skövde, 541 28 Skövde, Sweden

## Abstract

Paratuberculosis is a chronic bowel disease of ruminants caused by *Mycobacterium avium* subsp. *paratuberculosis* (MAP). Its secretion through semen highlights the importance of paratuberculosis-free breeding bulls. The breeding and teaser bulls at three semen production units (SPUs) located in Punjab, Pakistan, were screened for the presence of antibodies against MAP. A total of 253 samples were collected from SPUs and a commercially available indirect screen ELISA (Is-ELISA) was applied. Is-ELISA detected antibodies in 20 (24.6%), 16 (22.8%), and 17 (16.6%) samples from SPU-I, SPU-II, and SPU-III, respectively. Collectively, seroprevalence of 20.0% (47/235) in breeding bulls and 33.3% (6/18) in teaser bulls was observed, and thus it poses a potential threat of disease spread to a high number of heifers and cows through artificial insemination. Therefore, this paper highlights the presence of the disease for the first time at SPUs and triggers attempts to ascertain the prevalence of paratuberculosis throughout the country.

## 1. Introduction

Paratuberculosis or Johne's disease (JD) is a chronic granulomatous bowel disease of ruminants caused by *Mycobacterium avium* subsp. *paratuberculosis *(MAP), which is a slow-growing, acid-fast bacillus that depends upon mycobactin for growth [1]. JD is of high economic importance around the globe, causing an annual loss of $200 million in United States herds only [2]. It is primarily a disease of cattle, sheep, and goats, but MAP can cause disease in both domestic and wild ruminants and other animals including primates. Animals at younger age are more susceptible to the disease and normally do not develop clinical disease until three years of age [3]. In the subclinical phase of the infection, the bacteria are shed in feces and animals remain a potential source of spread in the herd while this shedding of the microorganism can be as high as 10^10^ organisms per gram of the feces in clinical cases [4]. The disease at its terminal stage is characterized by decrease in milk production, rapid weight loss due to diarrhea and dehydration, diffused edema, and infertility [5].

In humans, MAP is also implicated to cause Crohn's disease (CD) resulting in substantial morbidity. However, this assumption has limited acceptance because the link remains inconclusive for the role of MAP in the disease occurrence in human. Due to implication of MAP in CD and its ability to survive at pasteurization temperature in milk, it is the need of the time to control the disease in the source population [6].

Although, the postnatal fecal-oral transmission is the main route of infection but vaginal discharge, colostrums, and milk may serve as additional sources of spread. Artificial insemination and wildlife ruminants reservoirs are also implicated to be the sources for transmission and spread of MAP between and within herds [7, 8]. In advanced clinical disease, MAP was detected in the genitalia and in the semen of the infected bulls. Due to the ability of the organism to survive under antibiotic treatment and resist freezing while semen conservation in liquid nitrogen, paratuberculosis is a common intrauterine infection to occur [9, 10].

Johne's disease is endemic in cattle herds even in the most developed countries such as in The Netherlands, Austria, USA, and Belgium with a prevalence rate of 54%, 7%, 41%, and 18%, respectively. In Europe, Sweden is the only country which is maintaining the paratuberculosis-free status. In Pakistan, though paratuberculosis was reported in 1913 (about a century ago) from a military dairy farm at Lahore, there is limited information available on the national prevalence of paratuberculosis in domestic ruminants [11]. To our knowledge, there is no accessible information regarding prevalence of the disease in governmental livestock and breeding units despite the fact that breeding bulls can be a potential source of MAP spread throughout the country.

Therefore, this study was designed to assess the presence of paratuberculosis for the first time at governmental breeding unit employing a commercial ELISA, which is a cost-effective alternative to culturing of organisms, and is accepted for paratuberculosis diagnosis, worldwide [12–14].

## 2. Materials and Methods

The veterinary practitioners collected the blood samples from all selected breeding bulls (*n* = 253) including 18 teasers from semen production units (SPUs) from Punjab, Pakistan. Sampling from these units was permitted on the condition to not disclose the actual name of the any of SPUs. Therefore, SPU-I, SPU-II, and SPU-III were used for three SPUs. The serum was separated and was collected in sterile screw-capped plastic vials and was held at −20°C until analysis. None of the animals was known to have been vaccinated against paratuberculosis or to having detectable history of the disease before sampling. To detect the antibodies against *Mycobacterium avium* subsp. *paratuberculosis* all the samples were subjected to paratuberculosis indirect screen ELISA, made available by Innovative Diagnostics, France. This assay developed according to the recommendation of OIE [15]. The test was performed according to the instructions described by the manufacture. Briefly, samples were neutralized in the buffer containing *Mycobacterium phlei* and were then loaded to the ELISA plate where each well was coated with anti-MAP epitopes. The formation of positive antibodies-antigen complex was conjugated with anti-ruminants IgG-peroxidase. This antibodies-antigen-conjugate complex was visualized by the addition of TMB (3,3′, 5,5′-tetramethylbenzidine) substrate after removing the excess of conjugate. Then, 100 *μ*L of stop solution was added to each well and was mixed by tapping. The absorbance values (OD) were read immediately (within 10 minutes) at 450 nm.

The S/P percentage was calculated using the following formula:

(1) $$\text{\%}\frac{\text{S}}{\text{P}}={\text{OD}}_{\text{S}}-\frac{{\text{OD}}_{\text{NC}}}{{\text{OD}}_{\text{PC}}}-{\text{OD}}_{\text{NC}}\times 100,$$

where S stands for sample, P stands for positive, NC for negative control, and PC for positive control. The samples with S/P percentage ≤60% were considered negative while S/P ≥70% were declared as positive. The samples having S/P percentage >60% and <70% were considered doubtful and retested.

## 3. Results

**Figure 1:**
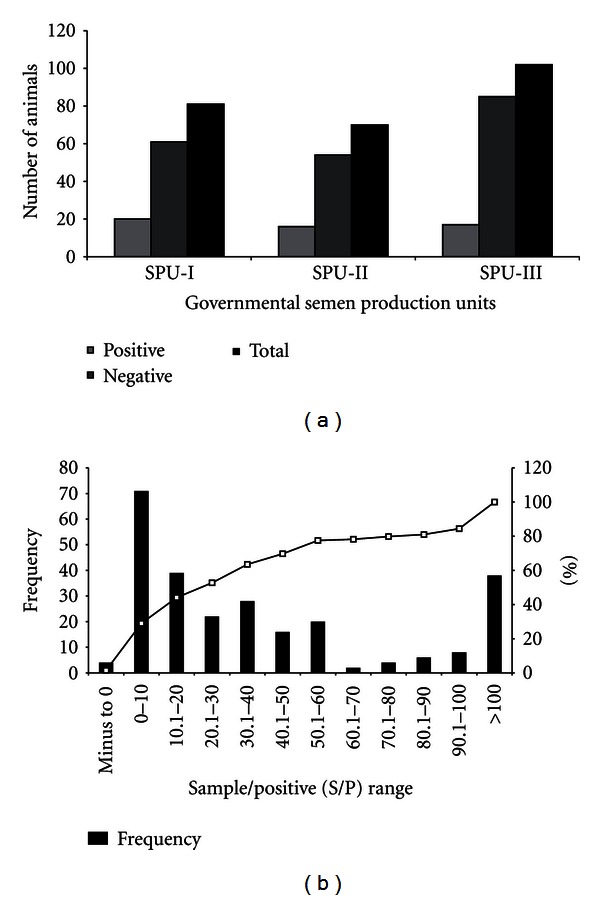
(a) Prevalence of paratuberculosis in breeding and teaser bulls. (b) Frequency distribution of paratuberculosis in breeding and teaser bulls.

Out of total samples tested, the overall rate of prevalence was 21.7% (*n* = 56) in the herd. This observed prevalence based on ELISA antibodies detection clearly differentiated the exposed (infected) population from the unexposed (not infected) population. Notably, the doubtful samples (carrying OD values from 60.1 to 70%) were not included in this prevalence. At SPU-I, of the 76 breeding bulls tested, 19 were found positive giving a seroprevalence of 25.0% while in teasers 1 out of 5 tested samples were observed to be positive giving a positive percentage of 20.0 (Figure 1(a)). The characteristics of the breeding and teaser bulls used in the study are presented in Table 1. Similarly, at SPU-II and SPU-III, a seroprevalence of 20.6% and 15.6% among breeding bulls was observed whereas 42.8% and 33.3% among teaser bulls was observed, respectively.

Among the samples considered negative for paratuberculosis (percent color inhibition of less than 60%) the greatest number of samples had a percent color inhibition of in between 1 to 10 followed by 10.1 to 20%. Alternatively, among the samples considered positive for paratuberculosis, (percent color inhibition of greater than 60%) a peak frequency distribution of 100% color inhibition was observed for both breeding and teaser bull population (Figure 1(b)).

## 4. Discussion

In this study, we identified and confirmed for the first time that the bulls are susceptible to Johne's disease at the semen production units (SPU) in Punjab, Pakistan. It is to mention that Punjab province is the largest province of Pakistan in terms of animal population and number of SPUs (4 SPUs are in Punjab out of total 8 governmental SPUs in the country). Therefore, it is a heavy load on these SPUs for semen production, and each breeding bull is supposed to produce 12000 doses per year. This preserved semen doses are then distributed to artificial insemination (AI) center throughout Punjab province. Given that the presence of MAP in semen of a naturally infected ram has been demonstrated [16], it is of paramount importance to ascertain the level of transmissible diseases in these SPUs because these can serve as a potential source of spread to the rest of the country where AI is practiced with this semen. Moreover, paratuberculosis is a matter of serious concern for not only livestock sector where it causes heavy economic losses, but also poses a risk to the public health. Although still controversial, the role of the organism in the etiopathology of human Crohn's disease is getting appreciation as recently bacterium has been isolated from the breast milk of a patient with Crohn's disease [17]. 

At SPU-I and SPU-II, it has been claimed to maintain the best hygienic conditions by giving showers at least once daily but the personnel involved in this management are poor and untrained, hence likely to perform semen collection under poor-hygienic conditions. This situation gets even worse at artificial insemination stations, where during insemination, contamination of semen by contaminated utensils and veterinary equipment leads to abortion and infertility [10]. This secondary contamination can be circumvented by stringent hygiene during collection of semen. Though all the bulls are kept in individual and separate pens (10 × 10 ft) in an open yard of 10 × 25 ft dimension, the personnel involved in management are shared between bulls and the rest of the flock which poses a continuous threat for the spread of disease.

Eradication of paratuberculosis is difficult due to long survival rate of the disease and insensitive diagnostic tests [18]. Moreover, because the MAP may go in latent stage (12 to 16 weeks) of infection and the level of detectable antibodies through ELISA are significantly reduced, the ability to identify a truly positive paratuberculosis remains limited. The sensitivity of the commercial ELISA has been estimated which ranged from 11% in early subclinical infections to over 90% in the terminal stages of disease with an average sensitivity of about 50% [19]. If the majority of the sampled bulls were culled because of lack of production and reproductive failure and not due to clinical Johne's disease, then the sensitivity of the ELISA could be assumed to be low. The total average life of breeding bull is calculated to be 12-13 years with 7-8 years of breeding life; therefore most of the trained bulls despite low production are kept at these SPUs.

The overall prevalence of disease in the country is lacking, despite the fact that paratuberculosis has been identified a century ago. This is primarily because of the long latency stage of the disease while it undergoes unnoticed, but lack of knowledge of strain diversity and readily available diagnostic test may also contribute to the disease being ignored. A study conducted by Khan et al. [20] indicates the presence of bacterium from the terminal ileum and mesenteric lymph node in 1000 cattle and buffalos slaughtered at an abattoir close to Lahore, without any precautionary measures. Most of the animals were emaciated and diarrheic, a sign of paratuberculosis [5]. However, it is hard to interpret from where these animals were bought and hence it is difficult to locate the region where disease is prevalent. This initiative to mark the identification of disease in SPU will help to increase the awareness for paratuberculosis and bring the attentions for government to take initiative for the prevalence and control of this disease in the country.

## 5. Conclusions

Because the present study is conducted on a limited number of samples from breeding and teaser bulls maintained at semen production units, it is difficult to reach a meaningful conclusion with respect to breeds, age, geographic location, and overall prevalence. However, it is indicative of prevalence of paratuberculosis antibodies in governmental SPUs. The prevalence of paratuberculosis infection in cattle and buffalo bulls and its serious impact on the livestock industry make it one of the most important infectious agents for livestock. Moreover, further systematic seroepidemiological and isolation studies are warranted to find out the actual status of paratuberculosis in SPUs and elsewhere in Pakistan. Thus, further steps can be taken to control this emerging disease.

## Figures and Tables

**Table 1:** Characteristics of the breeding and teaser bulls.

Location of the sample collection	Nature of bulls	Results			Age (range in years)	Ejaculation body	
		Positive	Negative	Total		Per week	Scoring
SPU-I	Breeding	19	57	78	3–11	2	6/10
	Teaser	1	4	5	3–11	—	7/10
SPU-II	Breeding	13	50	63	3–11	2	6/10
	Teaser	3	4	7	3–12	—	7/10
SPU-III	Breeding	15	81	96	3–10	2	8/10
	Teaser	2	4	6	3–11	—	7/10

Total	—	53	200	253	—	—	—
